# Analysis of Conventional and Enhanced-Biocompatibility ZnO/Ag Heterojunction Nanorod-Based Advanced Root Canal Sealers

**DOI:** 10.3390/bioengineering12090917

**Published:** 2025-08-26

**Authors:** Gayathri Velusamy, Aleena Unnikrishnan, Dinesh Veeran Ponnuvelu, Selvakumar Rajendran, Sungsu Park, Biji Pullithadathil

**Affiliations:** 1 Department of Conservative Dentistry and Endodontics, Sri Ramakrishna Dental College and Hospital, Coimbatore 641006, India; nvgayu@gmail.com; 2 Nanosensors & Clean Energy Laboratory, Department of Chemistry& Nanoscience and Technology, PSG Institute of Advanced Studies, Coimbatore 641004, India; aleenaunnikrishnan99@gmail.com; 3 School of Agricultural Sciences, Dhanalakshmi Srinivasan University, Tiruchirappalli 621112, India; 4 School of Mechanical Engineering, Sungkyunkwan University (SKKU), Suwon 16419, Republic of Korea; nanopark@skku.edu; 5 Department of Biotechnology & Nanobiotechnology, PSG Institute of Advanced Studies, Coimbatore 641004, India; selvabiotech@gmail.com

**Keywords:** root canal sealers, nano ZnO/Ag heterojunction, cytotoxicity, biocompatibility

## Abstract

This investigation aims to evaluate the biocompatibility and assess the cytotoxicity of synthesized ZnO/Ag heterojunction nanorods with commercially available root canal sealers in India. Among the commercially available root canal sealers, zinc oxide (ZnO) eugenol-based sealers are widely utilized as per Grossmann’s requirements. However, these ZnO eugenol-based sealers often experience solubility issues and tissue reactions in contact with periapical tissues. To overcome the inexplicable reactivity of ZnO eugenol-based sealers, nano ZnO and nano ZnO/Ag heterojunction materials have been developed via a wet-chemical approach and studied to assess their biocompatibility and cytotoxicity. The findings of our study revealed that nano ZnO/Ag heterojunction material possesses a higher degree of biocompatibility and low cytotoxicity as compared to conventional ZnO eugenol-based sealers, attributed to its high surface-to-volume ratio, the enhanced penetration of nanosized sealers into dentinal tubules, and the synergistic spillover sensitization effect of nano ZnO combined with Ag nanoclusters. From this comparative evaluation of root canal sealers, the usage of nano ZnO/Ag heterojunction materials was found to be significantly advantageous over commercial zinc oxide eugenol-based sealers and may find profound usage with a long shelf-life.

## 1. Introduction

Endodontic treatment is undertaken to inhibit and control infectious processes in the pulp and surrounding periradicular tissues, which is achieved through instrumentation, irrigation and intracanal medication. Nevertheless, these procedures do not eliminate the microorganisms from the root canal; hence, obturation of the root canal space with a suitable material that possesses antibacterial properties would be beneficial in further reducing the microbial load [1]. For endodontic procedures, materials should be judiciously selected based on comprehensive criteria that include their biocompatibility, ability to prevent leakage, and durability under intraoral conditions. The egress of chemical components from the obturation material through the apical foramen might lead to certain unfavorable periapical tissue reactions [2,3,4]. Extrusion of root canal filling materials into periapical tissues may provoke a localized bone tissue response, which is partly mediated by the activity of osteoblasts [3,4].

The essential properties of an ideal endodontic sealer encompass biocompatibility, dimensional consistency, effective sealing, and prolonged antimicrobial action [5,6]. Balancing physicochemical performance and biocompatibility in endodontic sealers is a persistent difficulty in material development. Achieving a balance between tissue compatibility and sealer performance remains difficult, as improvements in one property can lead to compromises in the other [7,8]. Zinc oxide eugenol (ZOE)-based sealers have traditionally been the most employed sealers during root canal treatment because they reasonably meet most of Grossmann’s requirements for sealers [5]. However, the drawbacks of the traditional zinc oxide eugenol sealer are its solubility and tissue reactions when it meets periapical tissues. Since their introduction, gradual developments in the chemistry and properties of ZOE sealers have been made to improve their quality.

Emerging research focuses on nanoparticle-enhanced root canal sealers, aiming to combine superior antimicrobial activity with improved sealing and mechanical characteristics [9,10]. Nanoparticles have attracted attention in endodontics because of their better penetration into the dentinal tubules, profound antibacterial properties and decreased micro-leakage [11,12]. Nanoparticles exhibit greater antibacterial activity because of their unique physicochemical properties, including high surface area and surface charge, that facilitate strong electrostatic interactions with bacterial cell membranes. Additionally, smaller particles have higher antibacterial activity than macroscale ones, which leads to them disturbing bacterial DNA replication through controlled ion release, leading to effective bacterial inactivation [13,14]. This multifaceted mode of action, combined with their ability to penetrate biofilms, positions nanoparticles as promising agents for improving antimicrobial efficacy in endodontic treatments [15,16,17].

The first tests of biocompatibility for any material to be used in patients are cytotoxicity studies. With respect to root canal sealers, the areas of potential tissue reaction are periapical tissues and bone. Therefore, assessing the biocompatibility of root filling materials on osteoblast-like cells, such as the MG63 cell line, is crucial to ensure their safe interaction with periapical bone tissue [18]. Cytotoxicity studies of root canal filling materials, conducted on MG63 human osteoblast-like cells, have given a fairly accurate picture of the biocompatibility of the material tested [19].

With this view, two experimental nano sealers—ZnO and ZnO/Ag heterojunction nanorods—were synthesized and tested for their biocompatibility in human osteoblast–G63 cells and further extended by evaluating their antimicrobial efficacy on the *Enterococcus faecalis* biofilm model grown on the root canals of extracted single-rooted teeth using confocal laser scanning microscopy against commercially available zinc oxide eugenol sealers. The present investigation aimed to evaluate and assess the biocompatibility and cytotoxicity of the synthesized ZnO/Ag heterojunction nanorods compared with the commercially available root canal sealers in India.

## 2. Materials and Methods

### 2.1. Synthesis of ZnO/Ag Heterojunction Nanorods

**Figure 1:**
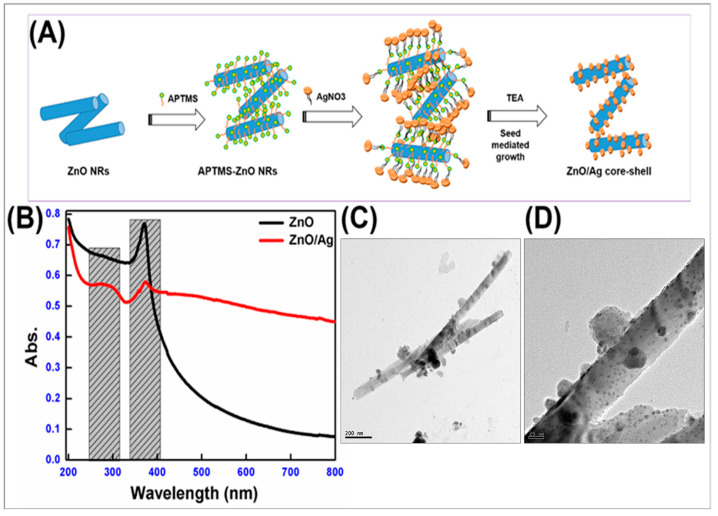
(**A**) Schematic representation of the synthesis of ZnO/Ag heterojunction nanorods. (**B**) UV–Visible spectrum of ZnO and ZnO/Ag heterojunction nanorods, and (**C**,**D**) TEM image of ZnO/Ag heterojunction nanomaterials.

The ZnO/Ag heterojunction nanorods used in the current study was prepared by adopting the procedure reported by Dinesh et al. [20]. Briefly, ZnO nanorod powders were prepared via the hydrothermal method using zinc chloride (ZnCl_2_), potassium hydroxide (KOH) and cetyltrimethylammonium bromide (CTAB) at a temperature regime of 120 °C for 5 h, followed by purification and drying to yield white-colored ZnO nano powders. Thus, the prepared ZnO nano powders were utilized to prepare ZnO/Ag heterojunction nanorods using the seed-mediated growth method using triethylamine (TEA) at 2–5 °C (Figure 1A). Commercial zinc oxide powders and eugenol (DPI, Mumbai, India) were used as the control materials. To confirm the nano structure of the synthesized materials, characterizations were performed via UV–Visible spectroscopy and Transmission Electron Microscopy (TEM) by using a T90+ UV–Visible spectrophotometer (PG Instruments, Lutterworth, UK) and a High-Resolution Transmission Electron Microscope (JEOL JEM-2010, Akishima, Japan), respectively.

### 2.2. Biocompatibility Testing

For biocompatibility testing, the osteoblast-like MG63 cells were cultured in Dulbecco’s modified Eagle medium (DMEM; Hyclone, Logan, UT, USA) supplemented with 10% fetal bovine serum (Hyclone) and antibiotics (100 µ/mL penicillin and 100 mg/mL streptomycin). The cultures were maintained at 37 °C in a humidified atmosphere with 5% CO_2_. A total of 10 × 10^3^ human osteoblastic cells MG63 were seeded into each well of a 96-well plate and incubated with DMEM (Dulbecco’s modified Eagle medium) at 37 °C and 5% CO_2_ overnight. Cells were treated with nano ZnO and nano ZnO/Ag heterojunction in the concentrations of 0.01 mg/mL, 0.05 mg/mL and 0.1 mg/mL for 24 h. A total of 20 μL MTT was added to each well and incubated for 3.5 h at 37 °C. The media was removed carefully from each well and 150 mL of DMSO (dimethyl sulfoxide) was added followed by agitation in an orbital shaker for 15 min. The optical density of each well was read at 590 nm using a 96-well microplate reader (Thermo Scientific™ Multiskan™ GO Microplate Spectrophotometer, Mumbai, India). The cell viability was estimated by comparing the absorbance of the cells cultured on different scaffolds to that of the control. MG63 cell viability was expressed as the percentage of optical density relative to the control. The results are presented as mean ± SD from two independent triplicate experiments.

### 2.3. Antimicrobial Studies

Antimicrobial efficacy studies on biofilm grown on the root canals of extracted single-rooted teeth using confocal laser scanning microscopy were performed for the 3 different groups, viz., ZnO eugenol sealer (commercial), nano ZnO eugenol sealer and nano ZnO/Ag heterojunction eugenol sealer; the detailed preparation methodology is discussed in Supplementary Material S1. To overcome solubility and tissue reactions while in contact with periapical tissues, zinc oxide (ZnO) eugenol-based sealers were upgraded to nano ZnO and nano ZnO/Ag heterojunction materials.

## 3. Results

### 3.1. Synthesis and Characterization of Nano ZnO and ZnO/Ag Heterojunction Nanorods

Nano ZnO and ZnO/Ag heterojunction nanorod-based sealers were synthesized using a wet-chemical method and characterized for optical and morphological confirmation using UV–Visible spectroscopy and Transmission Electron Microscopy, respectively. The ZnO nanorods showed a characteristic peak at 370 nm corresponding to the ground excitonic peak of pure ZnO nanorods on UV–Visible spectroscopic studies (Figure 1B). The ZnO/Ag heterojunction showed the existence of an excitonic peak (360 nm) along with characteristic surface plasmon peaks corresponding to Ag nanoislands at 270 nm and 420 nm [21,22]. High-Resolution Transmission Electron Microscopic analysis was employed to examine the morphological and structural properties of ZnO/Ag heterojunction nanorods. Figure 1C,D shows the TEM images of ZnO/Ag heterojunction nanorods with a clear overview of decorated Ag on the ZnO rods. The high-resolution image of heterojunction nanorods illustrates the uniform distribution of Ag nanoclusters on the surface of ZnO, corroborating the establishment of heterojunction layers. Lattice mismatch between wurtzite ZnO and cubic Ag facilitated the epitaxial growth of Ag nanoislands on ZnO surfaces [23]. Ag nanoclusters formed uniformly over the ZnO lattice without aggregation, averaging ~7 ± 0.5 nm in size, confirming the efficacy of the synthesis process.

### 3.2. Assessment of MG63 Cell Proliferation in Response to Material Eluates via MTT Assay

Synthesized nano ZnO and nano ZnO/Ag heterojunction sealer materials were checked for their biocompatibility using an MTT assay on human osteoblast-like MG63 cells against commercially available ZnO eugenol sealer. According to the methods reported in the ISO7405 (2025) [24] and the ISO10993-5 (2009) [25] standards (available online), there are basically two approaches to the in vitro evaluation of the cytotoxicity of experimental cements [26]. Either the material is directly placed in contact with cells, or the liquid extract of the material is placed in contact with cells [26,27]. In our study, we tested the material by placing it in direct contact with the cells. Per ISO 10993-5 (2009), a test material is deemed cytotoxic if its relative viability falls below 70%. This investigation quantified MG63 cell endurance post-exposure to graded eluate concentrations via MTT metabolic activity assay. The mean cell counts and percentage cell viability of MG63 cell lines at 24 h after treating with ZnO eugenol sealer (A), nano ZnO sealer (B) and nano ZnO/Ag sealer (C) at concentrations of 0.01 mg/mL, 0.05 mg/mL and 0.1 mg/mL compared with negative control (untreated cells) were recorded (Table 1).

**Figure 2:**
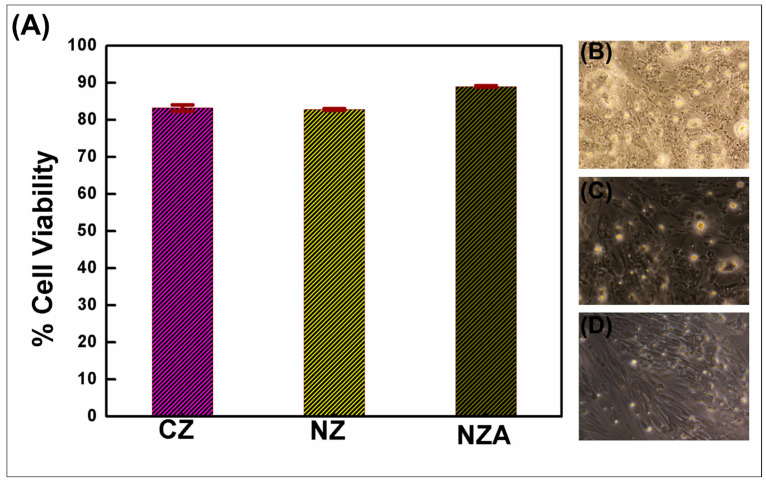
The effect of commercial zinc oxide, nano zinc oxide and nano zinc oxide silver heterojunction sealers at 0.1 mg/mL concentrations on the cell viability of MG63 cells (**A**), evaluated by the MTT test with their corresponding phase contrast microscopic images (**B**–**D**).

Figure 2 shows the effect of undiluted extracts of commercial ZnO sealer (A), nano ZnO sealer (B) and nano ZnO/Ag sealer (C) at three different concentrations of 0.01 mg/mL, 0.05 mg/mL and 0.1 mg/mL on the viability of MG63 cells, evaluated by the MTT test. Values are presented as the optical density percentage normalized to the negative control (100% viability). Cell viability for nano ZnO sealers was recorded at 98.27%, 86.23% and 82.70%, corresponding to exposures of 0.01, 0.05 and 0.1 mg/mL, respectively. At concentrations of 0.01, 0.05 and 0.1 mg/mL, nano ZnO/Ag heterojunction sealers exhibited cell viabilities of 95.45%, 90.68% and 88.91%, respectively (Table 1). Compared to the positive control—a commercial zinc oxide sealer exhibiting cell viabilities of 90.19%, 88.48% and 82.66% at 0.01, 0.05 and 0.1 mg/mL—both test materials demonstrated enhanced biocompatibility (Figure 2A).

The studies were further extended to analyze the cell damage using the MTT assay after exposing the cells to various concentrations of ZnO eugenol, nano ZnO and nano ZnO/Ag sealers (Figure 2B–D). The MTT assay showed no significant decrease in cell viability, suggesting that the nano sealers did not have any effect on cell toxicity. Over 88.91%, 82.70% and 82.6% cell viability was observed for nano ZnO/Ag, nano ZnO and commercial ZnO, respectively, for a 0.1 mg/mL concentration, which was confirmed by a live–dead cell assay. The response of cells to the nano sealers was directly measured by examining the activity of the mitochondrial enzyme succinate dehydrogenase (SDH).

### 3.3. Effects of Eluates on Antimicrobial Efficcacy Against E. faecalis in an Ex Vivo Tooth Model

**Figure 3:**
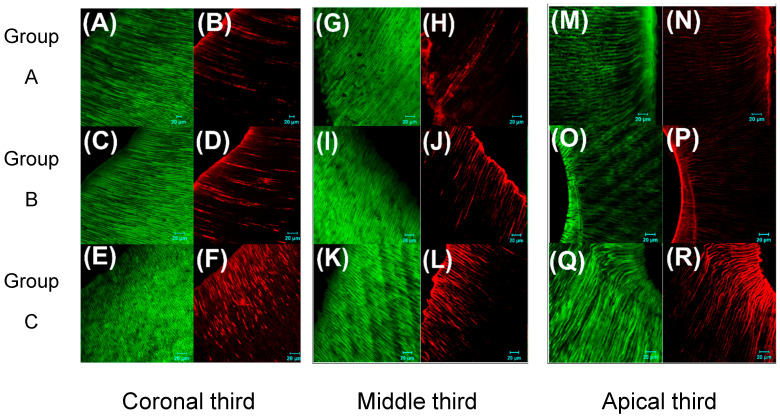
CLSM images for bacterial viability. Green fluorescence indicates live bacteria and red fluorescence indicates dead bacteria. Coronal third segment images of three groups (**A**–**F**), middle third segment images of three groups (**G**–**L**) and apical third segment images of three groups (**M**–**R**).

The nano ZnO and nano ZnO/Ag heterojunction sealers and the commercial ZnO eugenol sealers were tested for their antimicrobial efficacy against *E. faecalis* in root canals and incubated aerobically at 37 °C for 21 days. After incubation, the roots were embedded in self-cured acrylic resin and divided into transverse sections to obtain 42 sections/samples per group, and finally fluorescent-stained and visualized with a confocal laser scanning microscope (Figure 3). The observed CLSM images for the coronal third segment, middle segment and apical third segment clearly indicate that group C, treated with the nano ZnO/Ag heterojunction sealer, shows the best antimicrobial efficacy.

## 4. Discussion

The synthesized nano ZnO and nano ZnO/Ag heterojunction sealers were investigated for their optical and morphological properties. Optical studies of the nano ZnO/Ag clearly demonstrated the formation of heterojunctions with the presence of a modified ground excitonic peak (red-shift) of ZnO along with the surface plasmon peaks of Ag at 270 nm and 420 nm (Figure 1) [23]. The occurrence of heterojunctions was visually confirmed from the TEM image analysis with an average particle size of ~7 ± 0.5 nm Ag nanoclusters decorated over ZnO nanorods. The growth of Ag nanoclusters over ZnO occurred as a result of lattice mismatch, further enhancing the nucleation growth and thereby resulting in heterojunction formation, as evidenced by Figure 1C,D.

Comparative biocompatibility and cell viability evaluation of the nano ZnO sealers and nano ZnO/Ag heterojunction sealers compared to commercial ZnO eugenol sealers showed profound differences. Compared to the positive control group, zinc oxide eugenol (A), the experimental nano sealers, namely nano ZnO (B) and nano ZnO/Ag (C), showed better cell viability and hence less cytotoxicity/ better biocompatibility. The nano ZnO sealers and nano ZnO/Ag heterojunction sealers exhibited higher cell viability percentages for the 0.01 mg/mL, 0.05 mg/mL and 0.1 mg/mL concentrations. The greater cell viability of the nano sealers clearly affects the sealing activity, demonstrating higher efficiency and stability. Live–dead cell imaging implies the same level of compatibility owing to the higher surface-to-volume ratio, surface-bound atoms and favored electron transport chain from Ag nanoclusters to ZnO rods [23,26].

Root canal-based antimicrobial studies were performed against *E. faecalis* for 21 days, incubated aerobically at 37 °C. The nano ZnO/Ag sealer (C) showed significantly greater efficacy against *E. faecalis* than the nano ZnO sealer (B) and ZnO sealer (A) (*p* < 0.05), as detailed in Supplementary Tables S1–S3. Dead bacterial count showed a significant difference in the coronal and apical thirds in the two experimental groups compared to the conventional ZnO sealer group (Figure S2). The complete eradication of *E. faecalis* was not accomplished by any of the three root canal sealers tested. As reported earlier, the combination of chitosan nanoparticles and zinc oxide nanoparticles possesses a potential antibiofilm capability against *E. faecalis* [28,29]. Another study showed that nanoparticulate ZnO possessed a better antimicrobial effect against *E. faecalis* than microparticulate ZnO but worse efficacy than the combination of chlorhexidine with micro ZnO particles [30]. To further enhance the antibacterial properties of the ZnO nanoparticles, Ritika et al. modified the nanoparticles with silver nanoclusters using a biosynthetic method [31]. Incorporation of silver nanoparticles into endodontic sealer formulations enhanced antimicrobial efficacy without compromising the sealer’s flow properties [32]. Silver nanoparticles act as a sustained source of bioactive silver ions in aqueous conditions, leveraging a continuous ion release that underpins their potent antimicrobial function [32,33]. The increased antimicrobial potency of smaller silver nanoparticles arises from their expanded surface area, which promotes more effective engagement with bacterial cells compared to their larger counterparts [34]. Silver ions exert bactericidal effects by binding to essential enzymes and proteins, particularly their sulfhydryl (-SH-) groups, disrupting bacterial cell integrity [34,35]. Ag nanoparticles simultaneously target multiple microbial cell components—including the membrane, enzymes and plasmids—minimizing the bacteria’s potential to develop resistance. In a study, the antimicrobial effect of silver nanoparticle-based endodontic irrigants against E.faecalis, in comparison with NaOCl and chlorhexidine, showed that the silver nanoparticle-based irrigants were more efficacious than NaOCl and CHX [36,37]. This greater viability and biocompatibility arises from heterojunction formation with the spillover sensitization phenomenon of Ag nanoclusters inside the three-dimensional matrix of sealer materials owing to controlled stability [38].

Finally, from the present comparative investigation on the biocompatibility and bio-efficacy of dental sealer materials, nano ZnO/Ag heterojunction sealers showed higher efficacy compared to nano ZnO and commercial ZnO eugenol sealers against *E. faecalis*. This may be caused by the synergistic effect of nano zinc oxide and surface-doped Ag nanoclusters. Another factor that could have influenced the enhanced efficacy is the penetration ability of the nano sealers deep into the dentinal tubules compared to the traditional zinc oxide sealer, possibly due to their particle size effects and higher surface-to-volume ratio.

## 5. Conclusions

The findings of our study revealed that nano ZnO/Ag heterojunction material possesses a higher degree of biocompatibility and low cytotoxicity as compared to conventional ZnO eugenol-based sealers, due to its high surface-to-volume ratio, the high penetration ability of nanosized sealers deep into the dentinal tubules, and the synergistic effect of nano ZnO over Ag nanoclusters with a favored spillover sensitization effect. From this comparative evaluation of root canal sealers, the usage of nano ZnO/Ag heterojunction materials was found to be significantly advantageous over commercial zinc oxide eugenol-based sealers and may find profound usage with a long shelf-life. Further research regarding the depth of penetration of the sealer and its solubility would give a complete picture regarding its potential in biofilm eradication and may open the next paradigm for root canal sealer materials to be a profound commercial success.

## Figures and Tables

**Table 1:** Comparison of mean cell counts and percentage viability relative to negative control of MG63 cell lines after treating with ZnO sealer (A), nano ZnO sealer (B) and nano ZnO/Ag (C) sealer at concentrations of 0.01 mg/mL, 0.05 mg/mL and 0.1 mg/mL compared with negative control (untreated cells).

Group	Concentration (mg/mL)	Mean Count	% of Cell Viability
Control		0.3076	100.00
A	0.01	0.2774	90.19
	0.05	0.2721	88.48
	0.1	0.2542	82.66
B	0.01	0.3022	98.27
	0.05	0.2652	86.23
	0.1	0.2544	82.70
C	0.01	0.2936	95.45
	0.05	0.2789	90.68
	0.1	0.2735	88.91

## Data Availability

The original contributions presented in the study are included in the article/Supplementary Material, further inquiries can be directed to the corresponding authors.

## Supplementary Materials

The following supporting information can be downloaded at: https://www.mdpi.com/article/10.3390/bioengineering12090917/s1. Figure S1. (a) Tooth embedded in customized agar model and (b) Obturated tooth mounted in clear acrylic using putty mold. Figure S2. Graph shows the number of live and dead bacteria after treatment with the test materials Group A (ZnO sealers), Group B (Nano ZnO sealers) and Group C (Nano ZnO/Ag heterojunction sealers). Table S1: Descriptive statistics. Table S2: Post hoc analysis of Green bacteria across the three groups. Table S3: Post hoc analysis of Red bacteria across the three groups.
