# Supplementary material for: Analysis of Conventional and Enhanced-Biocompatibility ZnO/Ag Heterojunction Nanorod-Based Advanced Root Canal Sealers
Source: Bioengineering (Basel). 2025 Aug 26;12(9):917. doi: 10.3390/bioengineering12090917 (PMC12467648; doi:10.3390/bioengineering12090917)
Supplement: Supplementary file 1 [file bioengineering-12-00917-s001.zip › bioengineering-3497999-supplementary.pdf]

bioengineering

Communication

Profound convention and enhanced biocompatibility analysis of ZnO/Ag heterojunction nanorods based advanced root canal sealers

Gayathri Velusamy[1,2], Aleena Unnikrishnan[2,3], Dinesh Veeran Ponnuvelu[2,3,5]*, Selvakumar Rajendran[4], Sungsu Park[5] and Biji Pulithadathil[2]*

[1] Department of Conservative Dentistry and Endodontics, Sri Ramakrishna Dental College and Hospital, Coimbatore, INDIA.
[2] Nanosensors & Clean Energy Laboratory, Department of Chemistry& Nanoscience and Technology, PSG Institute of Advanced Studies, Coimbatore, INDIA.
[3] School of Agricultural Sciences, Dhanalakshmi Srinivasan University, Tiruchirappalli, INDIA.
[4] Department of Biotechnology & Nanobiotechnology, PSG Institute of Advanced Studies, Coimbatore, INDIA.
[5] School of Mechanical Engineering, Department of Biomedical Engineering, Department of Biophysics, Institute of Quantum Biophysics (IQB), Biomedical Institute for Convergence at SKKU (BICS), Sungkyunkwan University (SKKU), Suwon, 16419, Korea.
* Correspondence: Dinesh Veeran Ponnuvelu (vp.dinesh@gmail.com), Prof. P. Biji (bijuja123@yahoo.co.in)

Abstract: This investigation aims to evaluate the biocompatibility and cytotoxicity assessment of the synthesized ZnO/Ag heterojunction nanorods with the commercially available root canal sealer in India. Among the commercially available root canal sealers, zinc oxide (ZnO) eugenol-based sealers are widely utilized as per Grossmann's requirements. However, these ZnO eugenol-based sealers often endure solubility issues and tissue reactions in contact with periapical tissues. To overcome the inexplicable reactivity of ZnO eugenol-based sealers, nano ZnO and nano ZnO/Ag heterojunction materials via wet chemical approach have been developed and studied for its biocompatibility and cytotoxicity assessments. The findings of our study revealed that nano ZnO/Ag heterojunction material possesses higher degree of biocompatibility and low cytotoxicity as compared to conventional ZnO eugenol-based sealers owing to its high surface to volume ratio, high penetration ability of nano sized sealers deep into the dentinal tubules and synergistic effect of nano ZnO over Ag nanoclusters with favored spill over sensitization effect. From this comparative evaluation of root canal sealers, the usage of nano ZnO/Ag heterojunction materials was found to be significantly advantageous over commercial zinc oxide eugenol-based sealers and may find a profound usage point with long shelf-life.

Keywords: root canal sealers; nano ZnO/Ag heterojunction; cytotoxicity; biocompatibility

Supplementary Material

For tooth sample preparation and infection studies, forty-two single-rooted human mandibular premolars with closed apices, extracted for orthodontic reasons were used. The teeth were collected, cleaned, sterilized and stored according to OSHA guidelines. Each tooth was radiographed to confirm the presence of a single patent canal. The tooth specimens were sectioned below the cemento-enamel junction with a slow speed diamond disc under water cooling to obtain a standardized tooth length 12 mm (Figure A1). The canals were accessed, and initially a size #10 Stainless Steel (SS) K file was inserted into the canal until the file tip was just visible at the apical foramen. The working length (WL) was kept 1mm short of the apical foramen. The root canals were prepared using Protaper files (Dentsply Maillefer,Switzerland) upto size F3. The canals were irrigated with sodium hypochlorite between each instrument and 17% EDTA (Dentsply Maillefer, USA) as a final

irrigant to remove the smear layer. All the roots were then washed and stored in saline. All the prepared teeth were packed in suitable autoclave pouches and autoclaved at 121 °C for 15-20 minutes.

S.1. A customized model was assembled for each tooth for the subsequent bacterial inoculation and incubation. Molten Agar was expressed into three fourth of 1.5 mL micro-centrifugal tubes and allowed to cool. Once the agar reached the gel stage the teeth were embedded in the agar up to the junction of middle and coronal third of the root (Figure S1-a). After mounting each tooth in centrifugal tube, all tubes were placed in the stand ready for bacterial inoculation. The ampule containing *Enterococcus faecalis* (MTCC 439, Microbial type culture collection and Gene bank, Chandigarh) was wiped with alcohol and was opened in a biological safety cabinet designed to protect against inhalation of aerosols and to protect the ampule from external contamination. A 24-hour pure culture suspension of *E. faecalis* (MTCC 439, Microbial type culture collection and Gene bank, Chandigarh) was cultivated in Brain Heart Infusion broth. Each root was inoculated with *E. faecalis* by placing the suspension in the root canal of each tooth with a micropipette. Approximately 0.5µL of suspension was placed in every tooth sample. All specimens were incubated aerobically at 37º c for 21 days. Fresh BHI broth was supplemented into the root canals weekly to ensure viability of bacteria.

S.2. After 21 days of incubation the roots were removed from the incubator and each root was rinsed with 5 ml of saline for 1 minute. Teeth were randomly divided into three groups – ZnO eugenol sealer, nano ZnO sealer and nano ZnO/Ag sealer, with fourteen teeth per group. Root canals were dried with paper point size 30/.06. The powder liquid component of zinc oxide eugenol sealer was mixed according to the manufacturer's instruction. With the same powder liquid ratio, the experimentally synthesized and characterized nano ZnO and nano ZnO/Ag heterojunction particles were mixed with eugenol liquid and the sealers were applied with lentulo spiral. Obturation of the teeth was done with F3 size master cone by cold lateral compaction. The excess gutta percha was seared on the coronal aspect and the orifice was sealed with cavit. All these procedures were carried out in Laminar Air Flow Chamber sterilized by UV light. The obturated teeth were stored in humidity chamber (Figure 18) at 100% humidity and 37°C for two weeks allowing complete setting of the sealers.

S.3. The roots were embedded in self-cure acrylic resin in a putty mold (Figure S1-b) and each root was then transversely sectioned into coronal, middle and apical third sections using hard tissue microtome (Leica, Germany) to obtain three sections from each tooth. Now the experimental groups contained 42 sections/samples per group. Group A: Forty-two specimens obtained by sectioning coronal, middle and apical third roots treated with ZnO Eugenol sealer. Group B: Forty-two specimens obtained by sectioning coronal, middle and apical third roots treated with Nano ZnO sealer. Group C: Forty-two

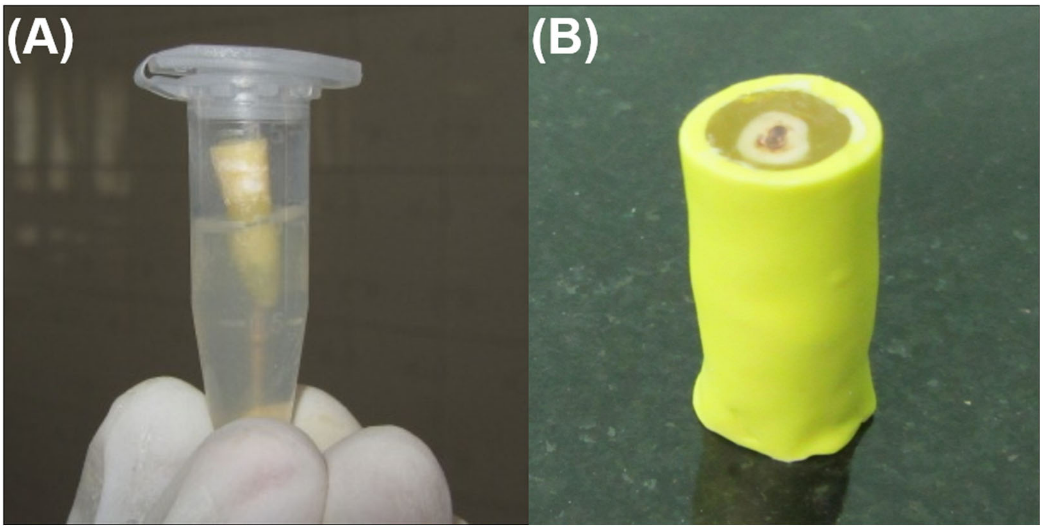

specimens obtained by sectioning coronal, middle and apical third roots treated with Nano ZnO/Ag heterojunction sealers.

Figure S1. (a) Tooth embedded in customized agar model and (b) Obturated tooth mounted in clear acrylic using putty mold.

S.4. The fluorescent dyes Fluorescein Diacetate diluted in acetone (FDA) and Propidium Iodide (PI) (Sigma, USA) diluted in distilled water were prepared to give a concentration of 4mg/mL of Fluorescein Diacetate and 1.4mg/mL of Propidium Iodide.

The root sections were washed with Phosphate buffered saline twice to remove any debris present. The root sections were placed in microcentrifugal tubes and 400 μL of FDA was added per vial in dark and the sections were kept immersed in the solution for 10 minutes at room temperature. Then the roots were removed from the vial and blotted dry and were immersed with PI for 2 minutes. All the sections were dried and were observed under a confocal laser scanning microscope (Zeiss, LSM 510 META, Germany) with 20X and 63X magnifications.

The fluorescent images obtained were in terms of green and red pixels, corresponding to live and dead bacteria. AIM software was used to assess the viability of *E.faecalis* against the various sealers used, by quantifying the bacteria individually as live and dead.

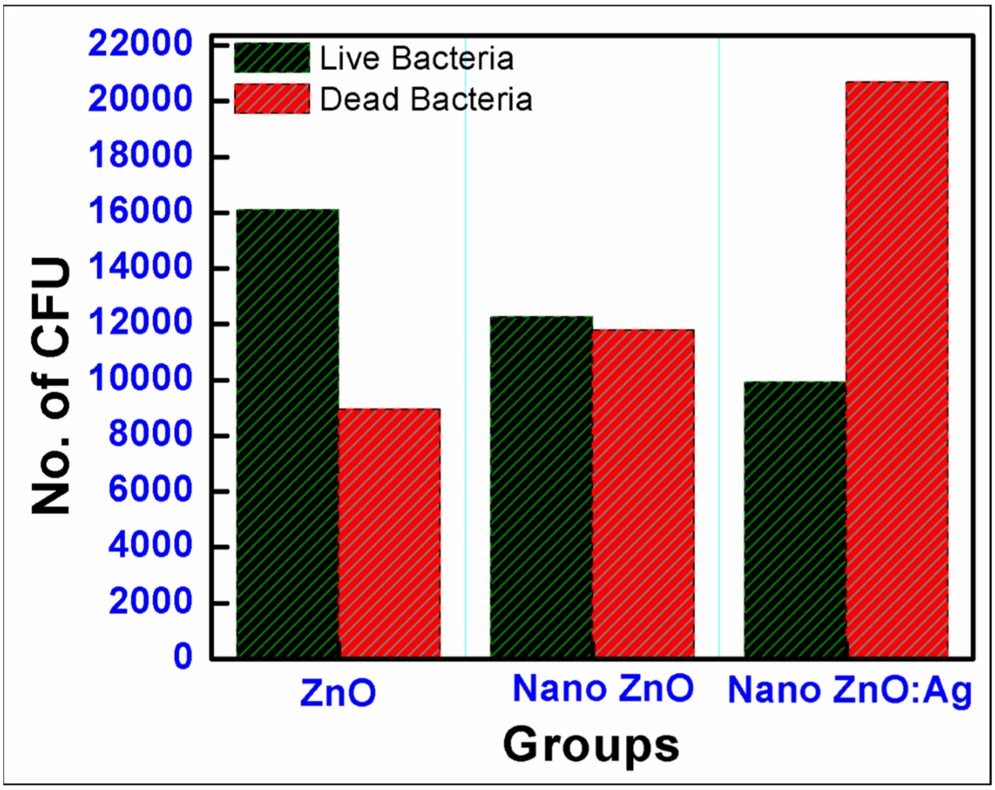

Figure S2. Graph shows the number of live and dead bacteria after treatment with the test materials Group A (ZnO sealers), Group B (Nano ZnO sealers) and Group C (Nano ZnO/Ag heterojunction sealers). The graph shows a smaller number of live bacteria and more number of dead bacteria in Group C compared to other groups.

The statistical analysis was performed with the SPSS 19 software system (IBM SPSS Statistics, Chicago, USA). Descriptive statistics was performed using One Way Anova followed by Tukey HSD (Post Hoc) with levels of significance set at $P < 0.05$.

Table S1: Descriptive statistics

Group	Cells	Minimum area (µm x µm)	Maximum area (µm x µm)	Mean	Standard deviation
(A) Control ZnO sealers	Live cells	6779	28307	16092.29	6174.849
	Dead cells	1598	20017	8940.43	4993.198
(B) Nano ZnO sealers	Live cells	1526	19953	12257.90	6267.430
	Dead cells	3828	34041	11801.00	7916.459
(C) Nano ZnO/Ag sealers	Live cells	2431	17158	9925.95	4195.413
	Dead cells	10775	45263	20689.33	8990.163

Table S2: Post hoc analysis of Green bacteria across the three groups

Group	Group	Mean difference	p value	95% CI	
				Lower bound	Upper bound
A	B	3834.381	0.078	-339.35	8008.11
	C	6166.333	0.002	1992.60	10340.06
B	C	2331.952	0.377	-1841.78	6505.68
**Significant p value is seen in Group C compared with Group A**					

Table S3: Post hoc analysis of Red bacteria across the three groups

Group	Group	Mean difference	p value	95% CI	
				Lower bound	Upper bound
A	B	-2860.571	0.436	-8417.58	2696.44
	C	-11748.91	0.000	-17305.92	-6191.89

B	C	-8888.333	0.001	-14445.35	-3331.32
**Significant p value is seen in Group C compared with Group A and Group B**					

Disclaimer/Publisher's Note: The statements, opinions and data contained in all publications are solely those of the individual author(s) and contributor(s) and not of MDPI and/or the editor(s). MDPI and/or the editor(s) disclaim responsibility for any injury to people or property resulting from any ideas, methods, instructions or products referred to in the content.
